# Height inequalities and their change trends in China during 1985–2010: results from 6 cross-sectional surveys on children and adolescents aged 7–18 years

**DOI:** 10.1186/s12889-017-4402-9

**Published:** 2017-05-18

**Authors:** Yong Xu, Lei Hang

**Affiliations:** School of Public Health, Medical College of Soochow University, No. 199 Ren Ai Road, Suzhou, 215123 People’s Republic of China

**Keywords:** Height, Inequalities, Changes

## Abstract

**Background:**

Great health inequalities have been reported in China over the past few years. Height has been used as an important parameter of health and it may also be distributed unequally in different regions. By studying height data of Chinese children and adolescents aged 7 to 18 years, we analyze height inequalities and their change trends during 1985–2010.

**Methods:**

On the base of data from 6 successive cross-sectional surveys of the Chinese National Survey on Student’s Constitution and Health(CNSSCH) conducted in 1985,1991,1995,2000,2005 and 2010, we calculated difference of height for children and adolescents aged 7–18 years in different regions. Coefficients of Variation (CVs) of height were computed in urban and rural areas during 1985–2010.

**Results:**

Great height difference existed between urban and rural, eastern and western, Shanghai and Guizhou children and adolescents aged 7–18 years. The urban-rural difference averagely decreased from 4.24 cm to 2.85 cm for boys and 3.72 cm to 1.31 cm for girls since 1985. Urban-rural difference tend to be more obvious in the poorer provinces, which has short mean statures. From 1985 to 2010, height difference became larger in eastern-western and Shanghai-Guizhou which represented the comparison between the richest and poorest regions. We also found there was a larger height inequality in rural areas compared with that in urban areas, and difference in rural subjects increased greater than their urban peers in eastern-western and Shanghai-Guizhou.

**Conclusions:**

There were obvious height inequalities in China and the urban-rural difference narrowed, while increasing differences happened between regions with different socioeconomic levels especially in their rural residents. More attention should be paid to these differences and policies and strategies should be developed to reduce inequalities in height.

**Electronic supplementary material:**

The online version of this article (doi:10.1186/s12889-017-4402-9) contains supplementary material, which is available to authorized users.

## Background

Height is an important indicator for growth and development because it not only reflects the nutrition and health condition in people’s childhood [1],but also influences their later life qualities and well-being. It is reported that taller people tend to have more superior outcomes by a variety of measures, such as productivity, income, longevity, life satisfaction and self-report health [2–6]. Height has been used as a proxy of the biological standard of living [7] and it can mirror the fluctuations of economic and epidemiological conditions of a country. It is a goal of every country to create a better environment for everyone to obtain his or her maximum genetic potentials in growth.

In the last few decades, China has great improvement in economy and public health [8] accompanied by a positive secular trend in the physical growth of children and adolescents [9–12]. However, these achievements were not distributed equally. In other words, increased income inequalities have emerged since the market reforms in 1978 [13] along with increased health inequalities [14–16]. Height of children and adolescents among different places also exerted strong heterogeneity in this period. A study on world height inequalities indicated that China existed greater inequalities in height compared with some other developing countries [17], suggesting that enough attention should be paid to this issue. Height inequalities highlight inequalities in health, nutrition and social well-being within populations and can provide important biological evidence for policy makers to develop some strategies in improving public welfare and equalities.

There was limited literature about height inequalities in Chinese children and adolescents. Chen TJ et al. [10] mentioned that height growth rate of children and adolescents aged 7–18 years varied among eastern, central and western rural regions during 1985–2010. In addition, Li H et al. [18] and Zong XN et al. [12] found that there was obvious height difference between urban and rural children aged 0–7 years. However, the fact that how height inequalities changed over time in the past few years remains unclear and the extent of difference in urban and rural areas as well as in regions with different socioeconomic levels needs to be further studied. We will address these issues in this study based on the national height data of Chinese students aged 7–18 years.

The purpose of this article is to examine height inequalities and their change trends from 1985 to 2010 with the measure of absolute difference. We will discuss in detail: (1) Whether there was urban-rural height difference and how it changed over the past 25 years. Whether height inequality in urban was greater than that in rural? (2) How height difference between eastern and western regions changed and how height difference between the richest (Shanghai) and poorest (Guizhou) provinces changed over time?

## Methods

### Data sources and subjects

The Chinese National Survey on Students’ Constitution and Health (CNSSCH) was the largest nationally representative sample of Chinese children and adolescents. It included a variety of constitution and health indicators such as height, weight, vital capacity and blood pressure. These data were reliable for researchers to estimate the growth and development situations of school-age boys and girls. CNSSCH was conducted systemically and successively in 1985,1991,1995,2000,2005 and 2010 [19–24]. Several government agencies had taken part in the surveys, including the State of National Affairs, the State Sports General Administration, the Ministry of Education, the Ministry of Science and Technology, and the Ministry of Health.

The subjects of this study were healthy children and adolescents aged 7–18 years from primary and middle schools, excluding those suffering from some diseases or physically handicaps. All subjects were selected with stratified cluster sampling from 28 to 30 mainland provinces. Each province had four subpopulations (urban male, urban female, rural male and rural female) which were stratified by areas of residence and sexes. The size samples of three socioeconomic classes (high, moderate and low) in each subpopulation were equal. To analyze the height difference between western and eastern regions, we divided these provinces into three regions according to their geographical locations and levels of economic development [25]. Eastern regions included Liaoning, Beijing, Tianjin, Shandong, Jiangsu, Shanghai, Zhejiang, Fujian and Guangdong. Western regions included Inner Mongolia, Guangxi, Shaanxi, Gansu, Ningxia, Qinghai, Xinjiang, Sichuan, Yunnan and Guizhou. Sample sizes for each sex-age subgroup were 16,596–17,122 in 1985, 5789–5949 in 1991, 8606–8762 in 1995, 8960–9183 in 2000, 9537–10,190 in 2005 and 8923–8992 in 2010, respectively (Additional file 1).

### Description and analysis

The height measurement was conducted by staff who had been trained specially following standardized methods and quality control procedures. Using metal column height measuring stand [26], heights were recorded to the nearest 0.01 cm. ‘Exact age’ was calculated to divide the subjects into 12 age groups. For example, the 7-year-old group (represented by ‘7+’) included children who were aged from 7 years to 1 month less than 8 years.

We use the measure of absolute difference which is sufficient for reporting inequality as it is straightforward and comprehensible [27]. T-test was used to analyze the differences in heights between urban and rural, eastern and western regions and Shanghai and Guizhou by the SPSS (PASW Statistics 18, SPSS Inc. Chicago, IL). To compare height equalities within urban and rural areas, coefficient of variation(CV) of height was computed in each sex-age group with the equation [28]: CV = (σ/μ)*100(σ and μ mean standard deviation and mean stature respectively).

## Results

### Urban-rural height difference and the inequalities within urban and rural

**Fig. 1:**
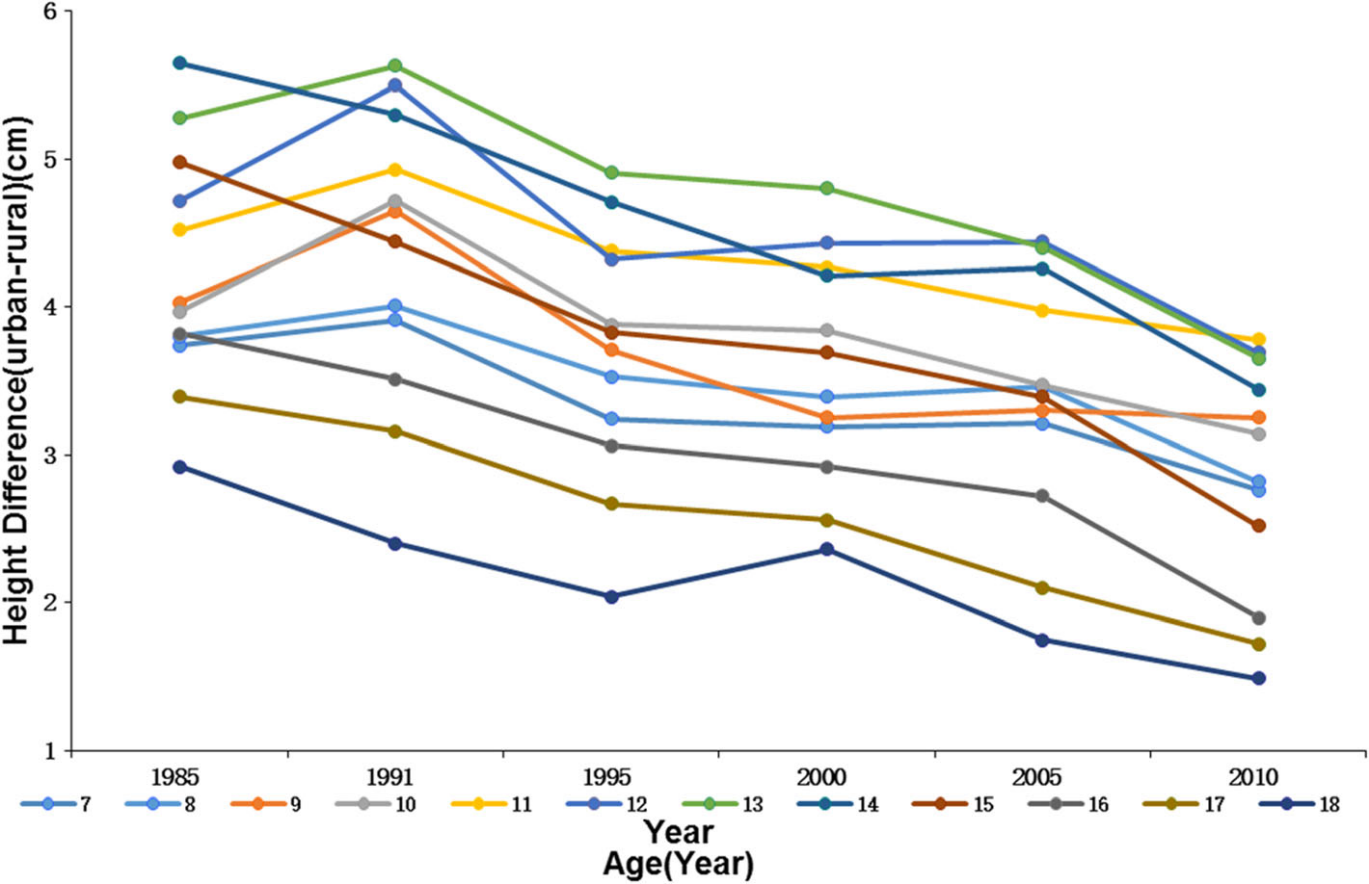
The height differences between urban and rural boys aged 7–18 years,1985–2010

**Fig. 2:**
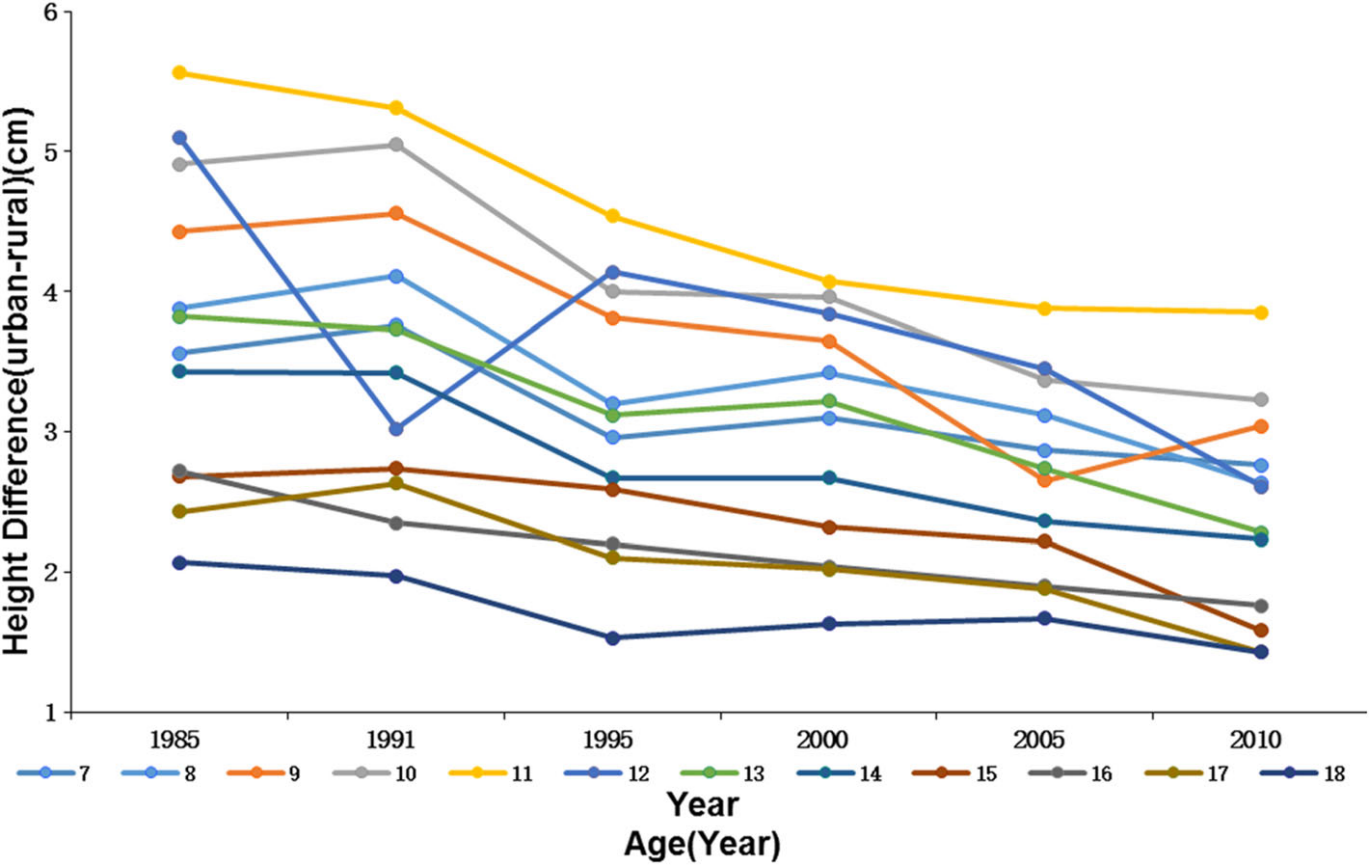
The height differences between urban and rural girls aged 7–18 years,1985–2010

Urban boys and girls were significantly taller (*P* < 0.001) than their rural counterparts in all age groups in both 1985 and 2010. For example, the height of urban boys and girls aged 7 years were 121.38 cm and 120.25 cm, and height of boys and girls in rural areas were 117.64 cm and 116.69 cm in 1985, respectively. The height differences between rural and urban kids were max for 14 years old boys and 11 years old girls (5.65 cm and 5.56 cm respectively) in 1985, whereas in 2010 the maximum differences were for 11 years in both boys (3.78 cm) and girls (3.85 cm), which might indicate an earlier age in peak height velocity along with time. From 1985 to 2010, urban-rural difference in height decreased continuously in most sex-age groups and the overall average decrease was 1.39 cm in boys and 1.31 cm in girls. We also found an interesting phenomenon that despite the large differences in height at younger ages, by age 18 the rural kids have nearly caught up, with only a 1.43 cm difference for boys (1.49 cm for girls) by 2010 compared with a 3.78 cm difference for boys aged 11 years(3.85 cm for girls). This is also true of the children in 1985 although the differences at age 18 were much larger (but nearly gone by 2010) (Figs. 1 and 2 **,** Additional file 2).

**Fig. 3:**
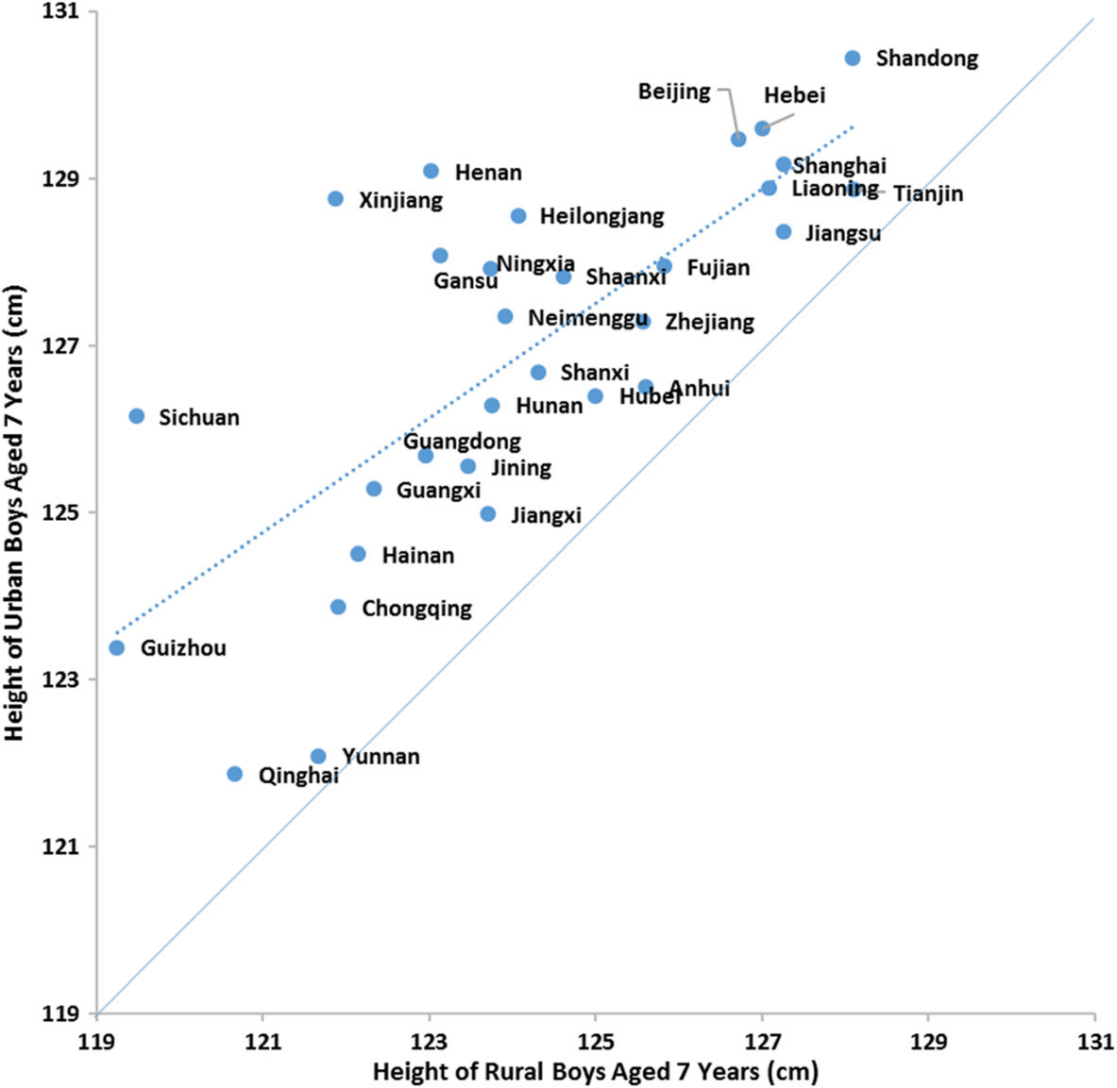
Height of urban and rural boys aged 7 years in different provinces in 2010

**Fig. 4:**
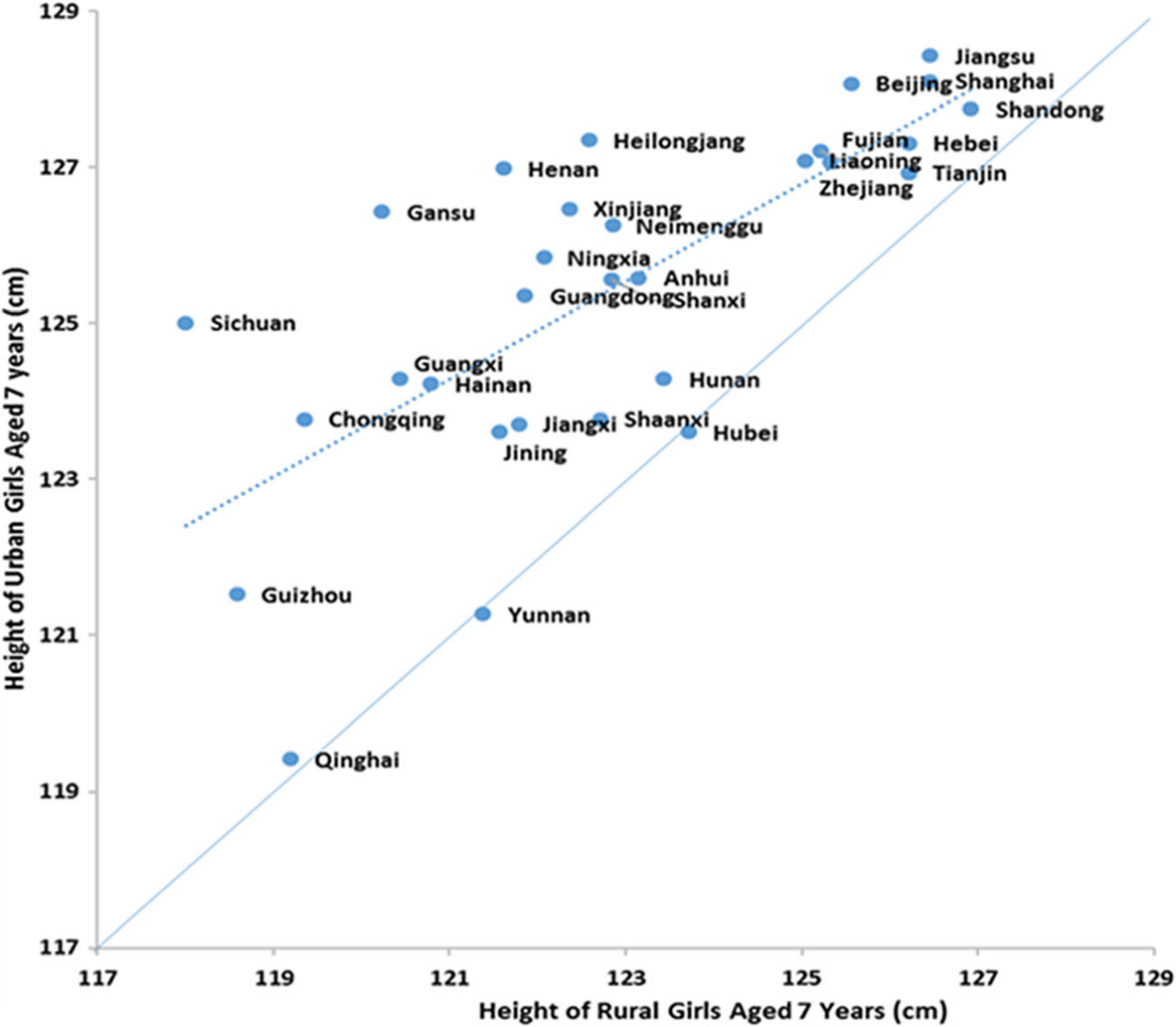
Height of urban and rural girls aged 7 years in different provinces in 2010

Figures 3 and 4 depicted the height of urban and rural children aged 7 years within each province in 2010. The points in the diagonal lines meant equal height between urban and rural areas and the farther away from the lines represented the larger urban-rural difference. From the trend lines, we can see that those provinces with shorter mean height tend to have greater urban-rural difference such as Guizhou, in which urban-rural difference was 4.14 cm and 2.95 cm for 7-year-old boys and girls, respectively, compared with that was 0.77 cm and 0.72 cm in Tianjin.

**Fig. 5:**
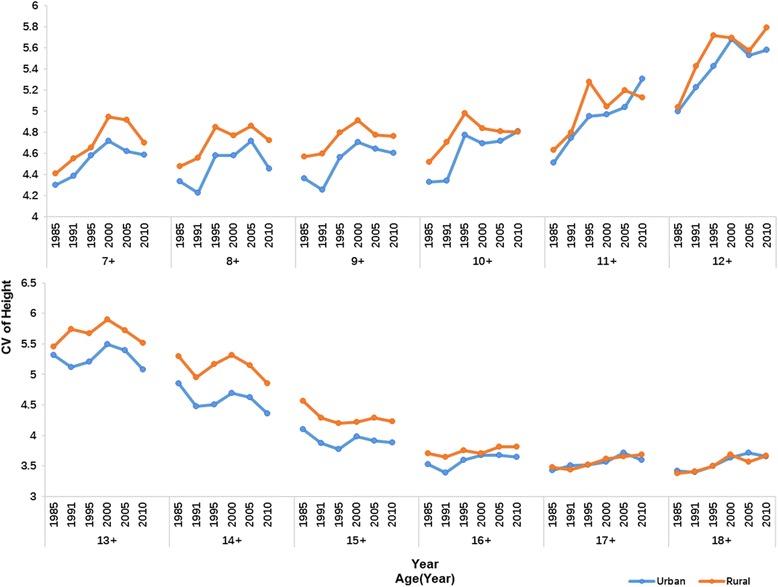
CVs of urban and rural boys’ height, 1985–2010

**Fig. 6:**
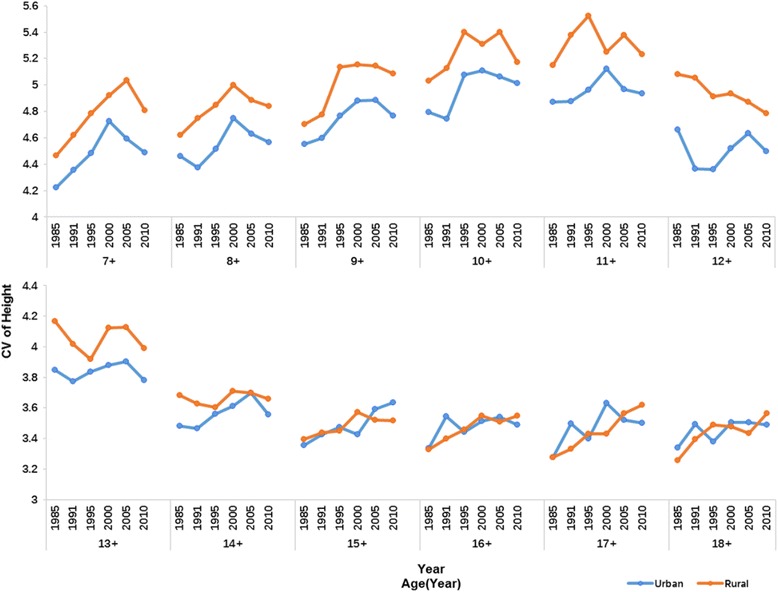
CVs of urban and rural girls’ height, 1985–2010

During 1985–2010, in almost all age specific groups, height CVs of rural boys and girls were larger compared to those of their urban counterparts and the differences were more obvious in smaller age groups. By age 18 years, CVs of height became very little in both urban and rural subjects. The variations in height increased over the 25 years in most age groups. For instance, the CVs increased from 4.3 to 4.58 for 7- year-old urban boys and 4.41 to 4.7 for rural boys. The exceptions were 13 to 15 years old boys and 12 to 14 years old girls whose CVs decreased over the past 25 years (Figs. 5 and 6).

### Eastern-western height difference

**Fig. 7:**
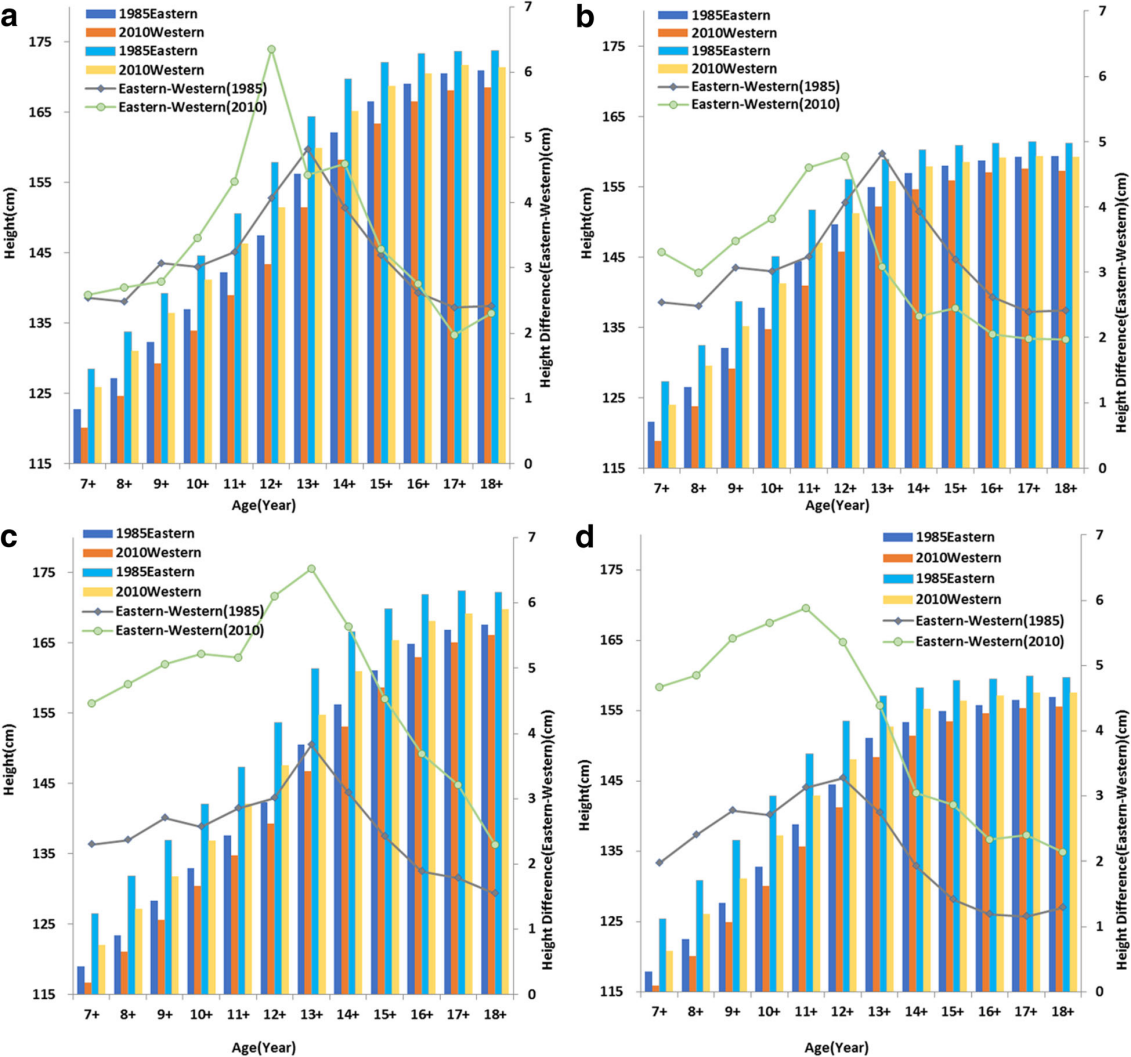
Mean height of Western and Eastern urban boys (**a**), urban girls (**b**), rural boys (**c**) and rural girls (**d**) aged 7–18 years and Western –Eastern differences in 1985 and 2010

**Fig. 8:**
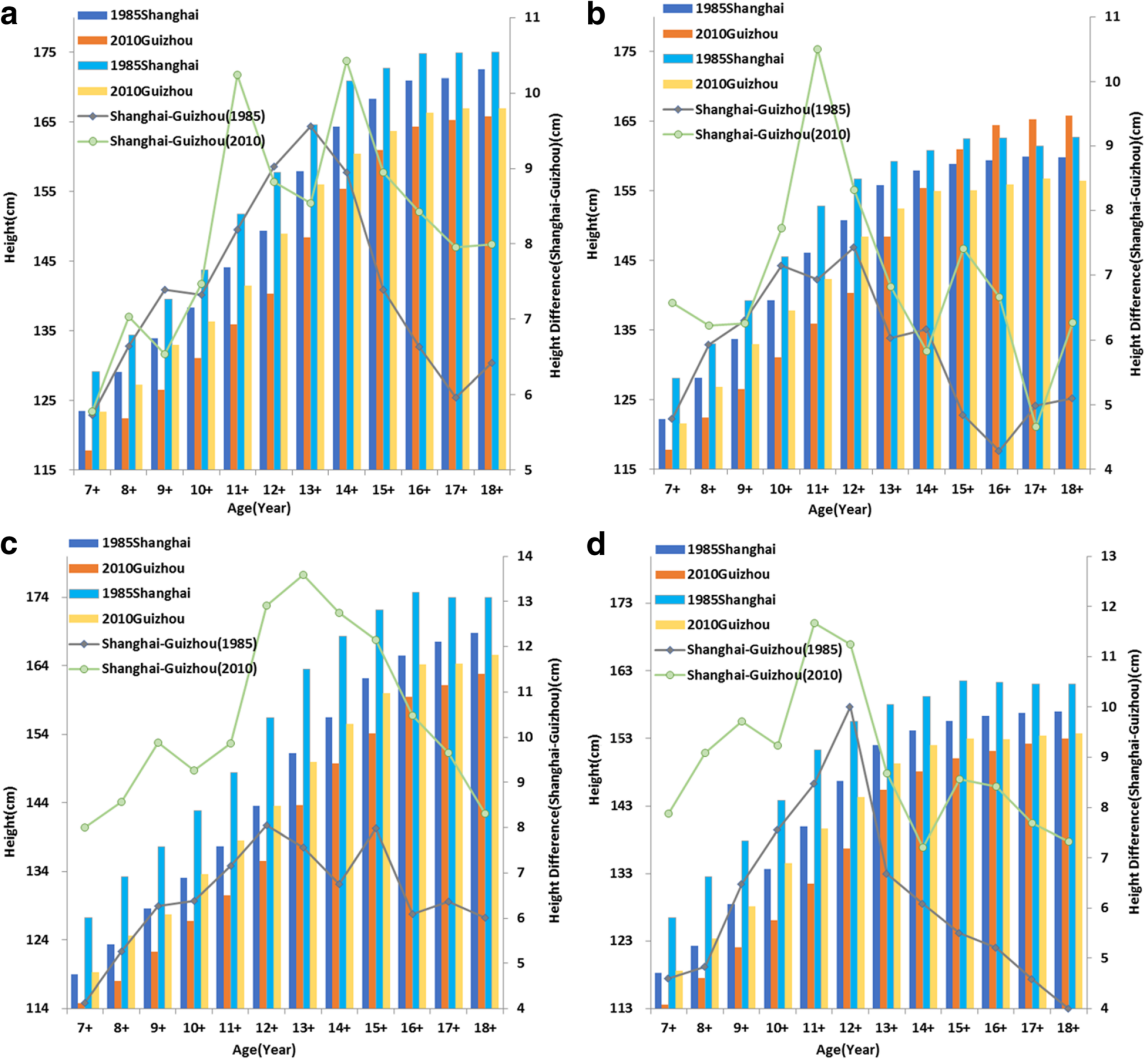
Mean height of Shanghai and Guizhou urban boys (**a**), urban girls (**b**),rural boys (**c**) and rural girls (**d**) aged 7–18 years and Shanghai–Guizhou differences in 1985 and 2010

In 1985 and 2010, eastern students of all 12 age groups were taller than their western peers both in urban and rural areas and all the differences had statistical significance (*P* < 0.001). The height differences between eastern and western kids were max for 13 years old boys (for 12 years old girls) in 1985 and by 2010, the age of largest eastern-western difference was 1 year earlier in urban children and rural girls but with no change in rural boys. Compared with 1985, eastern-western height difference in urban boys increased little for most age groups (maximum increase for 12 years old boys) in 2010. For urban girls aged over 13 years, eastern-western height difference even became smaller in 2010 than in 1985 (Fig. 8). While for rural boys and girls, the increase of eastern-western difference over the 25 years was obvious in all age groups with an increase of 2.2 cm for boys and 1.92 cm for girls averagely (Fig. 7 **,** Additional file 3).

### Shanghai-Guizhou height difference

In 1985, children and adolescents aged 7–18 years old who lived in Shanghai areas were significantly higher (*P* < 0.001) than those who lived in Guizhou areas with an average height difference of 7.43 cm (urban boys), 5.83 cm (urban girls), 6.5 cm (rural boys) and 6.17 cm (rural girls), respectively. Just like the height differences in urban-rural areas and eastern-western regions, the largest difference in Shanghai-Guizhou was also in puberty period. From 1985 to 2010, the differences between these two provinces changed little in urban boys and girls for most age groups but increased obviously by 10.45 cm for rural boys and 8.89 cm for girls in average (Fig. 8 **,** Additional file 4).

## Discussion

Those above results showed height inequalities in China from three dimensions and depicted how they changed over time by height data of children and adolescents among different age and sex groups.

As we all know, approximately 80% of individuals’ stature change is genetically or biologically regulated [29] and the remaining 20% is determined by environmental factors such as nutrition, diseases, living conditions and psychological stress, while genetic deviations cancel each other out from the average at the population level [30–33]. Therefore, it is plausible to speculate that not genetics but environment conditions related to socioeconomic factors are important determinants in height inequality.

Height is an appropriate indicator for measuring health inequality and health inequality is a priority for the post-2015 sustainable development goals (SDGs) [34]. The most obvious health inequality indicators such as mortality, morbidity, and life expectancy usually have measurement errors or reporting bias, but height does not suffer from these problems [35] and can be easily comparable across time and location with an objective scale. A large body of literature has investigated health conditions of children aged 0–17 based on the indicator of height-for-age-z-score (HAZ) [36–39]. In this study, we use mean stature data of children and adolescents aged from 7 to 18 years whose height were more sensitive to environment conditions to show the welfare and health inequalities in different areas and their change trends during 1985–2010.

The average urban-rural difference in height decreased from 4.24 cm to 2.85 cm for boys and 3.72 cm to 2.4 cm for girls during 1985–2010. From all the 24 sex-age groups, we found there was still an obvious height difference between urban and rural subjects although the difference decreased year by year. Huge disparity has existed for a long time between urban and rural areas not only in socioeconomic development but also in physical growth levels [18, 40]. It is generally recognized that the economic status of an area affects kids’ physical development by local infrastructure, sanitary conditions, nutrition quality and health-care access. Although income gap in urban and rural areas has deepened in the past few decades, our study indicates that height difference in these two areas has narrowed since 1985 and has a trend of decrease in the future. Our result was consistent with Chen’s study, which reported that rural children aged 7–18 years had a higher growth rate in stature than their urban counterparts [10] and it meant rural boys and girls were under their way to gain genetic potentials with improved rural living environment. On the other hand, urban children have been gradually moving toward the genetically determined upper limit of an individual’s potential for growth [9]. Thereby, children of older age groups in rural areas are catching up and shrinking the height difference with urban children by adequate supply of energy and nutrition. By age 18 years, the height gap between urban and rural kids narrowed to the least of all age groups in both 1985 and 2010.

The urban-rural differences varied in different provinces, for example, the average difference in Guizhou 7 years old boys was nearly 6 times larger than that in Tianjin. From the trend line in Figs. 3 and 4, we could find that the height differences between rural and urban children aged 7 years were relatively large in provinces where boys and girls had lower mean heights. This phenomenon was also seen in boys aged 2 to 3 months [41] and it might be explained by less access for nutrition and health resources with an unequal distribution of them in these provinces. It had been reported that there was a systematic negative and concave relationship between height inequality and average height [31]. However, some provinces such as Guizhou, Sichuan and Guangxi have great growth potentials for the high marginal returns, thus relevant polices should be formed to improve the living standard of these provinces so that children lived in these provinces can gain their genetic potentials.

It is evident that coefficient of variation (CV) is a widely used and robust index for measuring inequality [28] as it does not increase with average height like standard deviation (SD). Figures 5 and 6 show that CVs of rural subjects are higher than that of urban subjects in most age groups except 17–18 years old boys and 15–18 years old girls where rural CVs are similar to urban CVs. The corresponding explanation may be that rural children have a more unequal opportunity for necessities that are needful for growth such as food and medicine in contrast to their urban peers. Several studies have reported that urban areas exert lower extent of income inequality than rural areas [42] which may be related to the greater height inequality in rural area. Uneven distribution of height within rural areas was also found during 1975–1992 [41].Boys and girls at older age groups have a slower growth rate and relatively stable height with similarly little variations in urban and rural areas. From 1985 to 2010, height inequalities measured by CVs increased at most age groups with the exceptions of 13 to 15 years old boys and 12 to 14 years old girls. The figures show that maximal variation of height (CV) is around puberty period and it is very low when this period ends. This phenomenon can be related with an earlier age of growth peak over 25 years period. It was reported that the maximum height difference with the former age occurred in 13 years old boys and girls in 1985,while it changed to 12 and 10 years old for boys and girls in 2010, respectively [9]. Menarche age has been used for measuring tempo and adolescent maturation in a population and a marked secular trend in menarche has occurred in China since 1985. The average age at menarche for urban girls was 13.08 years in 1985, and it had fallen to 12.35 years with a decreasing rate of 3.5 months/decade in 2010. The decreasing rate of menarche age was even larger in rural girls with a rate of 5.8 months/decade (from 13.79 years to 12.59 years during 25 years period) [19 22]. The faster a population matures, the earlier the kids start their growth spurt and stop earlier [43].Height variation also decreases earlier in puberty period with the earlier end of growth peak. Therefore, it is natural that the CVs of height are decreasing during puberty period over study period. It is said that China has experienced high speed of economic development as well as increasing inequalities in the last few decades. The Gini coefficient which reflects economic inequality is 0.53–0.55 in 2010 (nearly twice the value of 1980s) [13], so China has a higher level of inequality than United States [44]. Previous research on British children found that there was a social gradient in height whereby children from poor groups had a lower average height than their more affluent peers [45]. Nevertheless, how income inequalities affect children and adolescents’ height in China is still unclear. We compared the height between western and eastern students as well as Shanghai and Guizhou students aged from7 to 18 years. It is well documented that for a long time eastern regions have higher level and speed of economic development than western regions with a wider income gap between these two regions [46]and significant differences are found between them by health outcomes such as infant mortality rate (IMR), maternal mortality rate (MMR) and under 5 mortality rate (U5MR) [47]. Shanghai is a modern metropolis with the highest per capita GDP every year in China since 1985, but Guizhou is a poor place where socioeconomic level lags behind all other provinces. Just as income difference in eastern-western regions and Shanghai-Guizhou provinces, significant height differences were found between these places in all sub-groups from 1985 to 2010. The largest eastern-western difference and Shanghai-Guizhou difference was found in puberty period and the age of maximum difference was earlier in 2010 compared with 1985, which can be explained by the earlier onset of peak growth in China as we mentioned before. We also found that there was an obvious increasing trend of the eastern-western height differences in rural subjects over the 25 years. While urban subjects in these areas did not have similar trends as the difference did not increase obviously and even decreased for some age groups in the past few years and it might indicate that the economic growth speed and physical growth rate in western urban region are similar to those in eastern urban region. This phenomenon was also seen in Shanghai and Guizhou urban regions. So rural students in western region and Guizhou province should be paid more attention in making polices to narrow height inequalities in China.

### Advantages and limitations of this study

This study use data from national surveys that are reliable and representative. In horizontal level, we explore the inequalities in height from three dimensions namely urban-rural areas, two regions and two provinces with different socioeconomic level and we research the change of height equalities along time in longitudinal level. Some limitations of our study should be discussed here. First, CNSSCH data began in 1985 and we could not explore the extent and change of height inequalities in a wider time range. Second, we analyzed the role of socioeconomic level of residence in children and adolescents’ height, but the data of some potentially important determinants of growth such as parental height, parental education level and birth height were lacking. Therefore, how these determinants influence and what extent they can explain the height inequalities in different regions remained unclear.

## Conclusions

In summary, our study found great height difference existed between urban and rural, eastern and western, Shanghai and Guizhou children and adolescents aged 7–18 years. Although the urban-rural height difference narrowed year by year, rural areas had greater inequality with uneven distribution of resources for children. The differences in eastern-western and Shanghai-Guizhou students’ height increased obviously from 1985 to 2010, especially in their rural subjects. Therefore, we should put rural areas in the priority place when making some policies and public health interventions. In order to shrink height inequalities and attain equal well-being for everyone in China, more equal opportunities for economic development as well as nutrition and health services should be given to different places especially in rural western regions and deprived provinces like Guizhou.

## Additional files

Additional file 1: Table S1. Sample sizes of subjects in urban and rural areas by different sex-age subgroups during 1985–2010 (DOCX 20 kb).

Additional file 2: Table S2. Height of urban and rural subjects and urban-rural height differences in 1985 and 2010 (DOCX 20 kb).

Additional file 3: Table S3. Height of eastern and western subjects and eastern-western height differences in 1985 and 2010 (DOCX 22 kb)

Additional file 4: Table S4. Height of Shanghai and Guizhou subjects and Shanghai-Guizhou height differences in 1985 and 2010 (DOCX 22 kb).

## Abbreviations

CNSSCH: Chinese National Survey on Student’s Constitution and Health
CV: Coefficient of Variation
HAZ: height-for-age-z-score
IMR: infant mortality rate
MMR: maternal mortality rate
SD: standard deviation
SDGs: Sustainable Development Goals
U5MR: under 5 mortality rate
