# Supplementary material for: Height inequalities and their change trends in China during 1985–2010: results from 6 cross-sectional surveys on children and adolescents aged 7–18 years
Source: BMC Public Health. 2017 May 18;17:473. doi: 10.1186/s12889-017-4402-9 (PMC5437404; doi:10.1186/s12889-017-4402-9)
Supplement: Supplementary file 1 — Sample sizes of subjects in urban and rural areas by different sex-age subgroups during 1985–2010 (DOCX 20 kb). [file 12889_2017_4402_MOESM1_ESM.docx]

Additional file 1: Table S1. Sample sizes of subjects in urban and rural areas by different sex-age subgroups during 1985-2010.

|  | 1985 | | | 1991 | | | 1995 | | | 2000 | | | 2005 | | | 2010 | | |
| --- | --- | --- | --- | --- | --- | --- | --- | --- | --- | --- | --- | --- | --- | --- | --- | --- | --- | --- |
| Age | Urban | Rural | Total | Urban | Rural | Total | Urban | Rural | Total | Urban | Rural | Total | Urban | Rural | Total | Urban | Rural | Total |
| Boys |  |  |  |  |  |  |  |  |  |  |  |  |  |  |  |  |  |  |
| 7+ | 8560 | 8546 | 17106 | 2933 | 2884 | 5817 | 4410 | 4348 | 8758 | 4446 | 4609 | 9055 | 4935 | 4817 | 9752 | 4486 | 4487 | 8973 |
| 8+ | 8561 | 8558 | 17119 | 2999 | 2950 | 5949 | 4398 | 4344 | 8742 | 4546 | 4541 | 9087 | 4934 | 4791 | 9725 | 4485 | 4485 | 8970 |
| 9+ | 8557 | 8556 | 17113 | 2959 | 2972 | 5931 | 4397 | 4348 | 8745 | 4571 | 4447 | 9018 | 4945 | 4814 | 9759 | 4481 | 4490 | 8971 |
| 10+ | 8557 | 8560 | 17117 | 2932 | 2894 | 5826 | 4390 | 4355 | 8745 | 4587 | 4542 | 9129 | 4916 | 4958 | 9874 | 4490 | 4490 | 8980 |
| 11+ | 8561 | 8561 | 17122 | 2965 | 2919 | 5884 | 4396 | 4354 | 8750 | 4524 | 4486 | 9010 | 5051 | 4818 | 9869 | 4500 | 4490 | 8990 |
| 12+ | 8558 | 8561 | 17119 | 2957 | 2911 | 5868 | 4398 | 4340 | 8738 | 4523 | 4502 | 9025 | 4917 | 4800 | 9717 | 4483 | 4496 | 8979 |
| 13+ | 8558 | 8557 | 17115 | 2906 | 2920 | 5826 | 4394 | 4347 | 8741 | 4546 | 4360 | 8906 | 4914 | 4763 | 9677 | 4487 | 4491 | 8978 |
| 14+ | 8560 | 8559 | 17119 | 2948 | 2919 | 5867 | 4403 | 4346 | 8749 | 4546 | 4459 | 9005 | 4852 | 4814 | 9666 | 4489 | 4496 | 8985 |
| 15+ | 8556 | 8559 | 17115 | 2952 | 2962 | 5914 | 4403 | 4344 | 8747 | 4561 | 4479 | 9040 | 4979 | 4951 | 9930 | 4489 | 4490 | 8979 |
| 16+ | 8559 | 8557 | 17116 | 2936 | 2959 | 5895 | 4343 | 4339 | 8682 | 4551 | 4470 | 9021 | 4892 | 4912 | 9804 | 4476 | 4486 | 8962 |
| 17+ | 8532 | 8560 | 17092 | 2936 | 2962 | 5898 | 4395 | 4334 | 8729 | 4507 | 4403 | 8910 | 4919 | 4871 | 9790 | 4488 | 4466 | 8954 |
| 18+ | 8324 | 8523 | 16847 | 2968 | 2965 | 5933 | 4335 | 4319 | 8654 | 4549 | 4546 | 9095 | 4979 | 5138 | 10117 | 4476 | 4490 | 8966 |
| Total | 102443 | 102657 | 205100 | 35391 | 35217 | 70608 | 52662 | 52118 | 104780 | 54457 | 53844 | 108301 | 59233 | 58447 | 117680 | 53830 | 53857 | 107687 |
| Girls |  |  |  |  |  |  |  |  |  |  |  |  |  |  |  |  |  |  |
| 7+ | 8559 | 8538 | 17097 | 2925 | 2887 | 5812 | 4400 | 4346 | 8746 | 4512 | 4493 | 9005 | 4860 | 4764 | 9624 | 4481 | 4493 | 8974 |
| 8+ | 8561 | 8560 | 17121 | 2977 | 2911 | 5888 | 4406 | 4356 | 8762 | 4533 | 4483 | 9016 | 4862 | 4746 | 9608 | 4469 | 4495 | 8964 |
| 9+ | 8561 | 8561 | 17122 | 2951 | 2904 | 5855 | 4402 | 4334 | 8736 | 4494 | 4515 | 9009 | 4892 | 4751 | 9643 | 4490 | 4495 | 8985 |
| 10+ | 8559 | 8561 | 17120 | 2942 | 2866 | 5808 | 4402 | 4357 | 8759 | 4657 | 4505 | 9162 | 4932 | 4836 | 9768 | 4487 | 4493 | 8980 |
| 11+ | 8559 | 8559 | 17118 | 2935 | 2936 | 5871 | 4413 | 4346 | 8759 | 4498 | 4487 | 8985 | 4894 | 4819 | 9713 | 4496 | 4496 | 8992 |
| 12+ | 8557 | 8562 | 17119 | 2875 | 2921 | 5796 | 4397 | 4350 | 8747 | 4518 | 4514 | 9032 | 4785 | 4752 | 9537 | 4484 | 4499 | 8983 |
| 13+ | 8558 | 8557 | 17115 | 2933 | 2918 | 5851 | 4395 | 4310 | 8705 | 4530 | 4503 | 9033 | 4925 | 4824 | 9749 | 4487 | 4482 | 8969 |
| 14+ | 8561 | 8556 | 17117 | 2931 | 2925 | 5856 | 4383 | 4344 | 8727 | 4513 | 4470 | 8983 | 4859 | 4763 | 9622 | 4494 | 4481 | 8975 |
| 15+ | 8556 | 8558 | 17114 | 2934 | 2926 | 5860 | 4394 | 4340 | 8734 | 4516 | 4454 | 8970 | 4935 | 4887 | 9822 | 4482 | 4495 | 8977 |
| 16+ | 8557 | 8557 | 17114 | 2911 | 2934 | 5845 | 4396 | 4272 | 8668 | 4519 | 4487 | 9006 | 4916 | 4830 | 9746 | 4454 | 4485 | 8939 |
| 17+ | 8537 | 8556 | 17093 | 2910 | 2916 | 5826 | 4403 | 4278 | 8681 | 4524 | 4459 | 8983 | 4857 | 4862 | 9719 | 4485 | 4486 | 8971 |
| 18+ | 8159 | 8437 | 16596 | 2933 | 2856 | 5789 | 4365 | 4241 | 8606 | 4632 | 4551 | 9183 | 5047 | 5143 | 10190 | 4435 | 4488 | 8923 |
| Total | 102284 | 102562 | 204846 | 35157 | 34900 | 70057 | 52756 | 51874 | 104630 | 54446 | 53921 | 108367 | 58764 | 57977 | 116741 | 53744 | 53888 | 107632 |
|  |  |  |  |  |  |  |  |  |  |  |  |  |  |  |  |  |  |  |
