# Supplementary material for: Height inequalities and their change trends in China during 1985–2010: results from 6 cross-sectional surveys on children and adolescents aged 7–18 years
Source: BMC Public Health. 2017 May 18;17:473. doi: 10.1186/s12889-017-4402-9 (PMC5437404; doi:10.1186/s12889-017-4402-9)
Supplement: Supplementary file 2 — Height of urban and rural subjects and urban-rural height differences in 1985 and 2010 (DOCX 20 kb). [file 12889_2017_4402_MOESM2_ESM.docx]

Additional file 2: Table S2 .Height of urban and rural subjects and urban-rural height differences in 1985 and 2010(cm)

|  |  |  | 1985 |  |  |  | 2010 |  |  |
| --- | --- | --- | --- | --- | --- | --- | --- | --- | --- |
|  | Age | Urban | Rural | d1 |  | Urban | Rural | d2 | d1-d2 |
| Male | 7+ | 121.38 | 117.64^*^ | 3.74 |  | 126.90 | 124.14^*^ | 2.76 | 0.98 |
|  | 8+ | 125.86 | 122.06^*^ | 3.80 |  | 132.15 | 129.33^*^ | 2.82 | 0.98 |
|  | 9+ | 130.88 | 126.85^*^ | 4.03 |  | 137.44 | 134.19^*^ | 3.25 | 0.78 |
|  | 10+ | 135.49 | 131.52^*^ | 3.97 |  | 142.45 | 139.31^*^ | 3.14 | 0.83 |
|  | 11+ | 140.53 | 136.01^*^ | 4.52 |  | 148.14 | 144.36^*^ | 3.78 | 0.74 |
|  | 12+ | 145.28 | 140.56^*^ | 4.72 |  | 154.23 | 150.54^*^ | 3.69 | 1.03 |
|  | 13+ | 153.66 | 148.38^*^ | 5.28 |  | 161.71 | 158.06^*^ | 3.65 | 1.63 |
|  | 14+ | 160.08 | 154.43^*^ | 5.65 |  | 166.99 | 163.55^*^ | 3.44 | 2.21 |
|  | 15+ | 164.78 | 159.80^*^ | 4.98 |  | 170.01 | 167.49^*^ | 2.52 | 2.46 |
|  | 16+ | 167.67 | 163.85^*^ | 3.82 |  | 171.48 | 169.58^*^ | 1.90 | 1.92 |
|  | 17+ | 169.24 | 165.85^*^ | 3.39 |  | 172.24 | 170.52^*^ | 1.72 | 1.67 |
|  | 18+ | 169.69 | 166.77^*^ | 2.92 |  | 172.17 | 170.68^*^ | 1.49 | 1.43 |
|  | Average |  |  | 4.24 |  |  |  | 2.85 | 1.39 |
| Female | 7+ | 120.25 | 116.69^*^ | 3.56 |  | 125.51 | 122.75^*^ | 2.76 | 0.80 |
|  | 8+ | 125.06 | 121.18^*^ | 3.88 |  | 130.72 | 128.09^*^ | 2.63 | 1.25 |
|  | 9+ | 130.52 | 126.09^*^ | 4.43 |  | 136.55 | 133.51^*^ | 3.04 | 1.39 |
|  | 10+ | 136.25 | 131.34^*^ | 4.91 |  | 142.87 | 139.64^*^ | 3.23 | 1.68 |
|  | 11+ | 142.52 | 136.96^*^ | 5.56 |  | 149.17 | 145.32^*^ | 3.85 | 1.71 |
|  | 12+ | 147.63 | 142.53^*^ | 5.10 |  | 153.46 | 150.85^*^ | 2.61 | 2.49 |
|  | 13+ | 153.38 | 149.56^*^ | 3.82 |  | 157.13 | 154.85^*^ | 2.28 | 1.54 |
|  | 14+ | 155.71 | 152.28^*^ | 3.43 |  | 158.91 | 156.68^*^ | 2.23 | 1.20 |
|  | 15+ | 156.77 | 154.09^*^ | 2.68 |  | 159.33 | 157.75^*^ | 1.58 | 1.10 |
|  | 16+ | 157.80 | 155.08^*^ | 2.72 |  | 159.91 | 158.15^*^ | 1.76 | 0.96 |
|  | 17+ | 158.18 | 155.75^*^ | 2.43 |  | 160.00 | 158.57^*^ | 1.43 | 1.00 |
|  | 18+ | 158.15 | 156.08^*^ | 2.07 |  | 159.91 | 158.48^*^ | 1.43 | 0.64 |
|  | Average |  |  | 3.72 |  |  |  | 2.40 | 1.31 |
|  |  |  |  |  |  |  |  |  |  |

T-test: rural compared with urban subjects, *P<0.001;

d1:height difference( urban -rural )in 1985;

d2:height difference (urban –rural )in 2010
