# Supplementary material for: Height inequalities and their change trends in China during 1985–2010: results from 6 cross-sectional surveys on children and adolescents aged 7–18 years
Source: BMC Public Health. 2017 May 18;17:473. doi: 10.1186/s12889-017-4402-9 (PMC5437404; doi:10.1186/s12889-017-4402-9)
Supplement: Supplementary file 3 — Height of eastern and western subjects and eastern-western height differences in 1985 and 2010 (DOCX 22 kb) [file 12889_2017_4402_MOESM3_ESM.docx]

Additional file 3: Table S3. Height of eastern and western subjects and eastern-western height differences in 1985 and 2010 (cm)

|  |  |  |  | Male |  |  |  |  |  |  |  | Female |  |  |  |  |
| --- | --- | --- | --- | --- | --- | --- | --- | --- | --- | --- | --- | --- | --- | --- | --- | --- |
| Urban | Age |  | 1985 |  |  | 2010 |  | d2-d1 |  |  | 1985 |  |  | 2010 |  | d2-d1 |
|  |  | Eastern | Western | d1 | Eastern | Western | d2 |  |  | Eastern | Western | d1 | Eastern | Western | d2 |  |
|  | 7+ | 122.69 | 120.15^*^ | 2.54 | 128.46 | 125.87^*^ | 2.59 | 0.04 |  | 121.55 | 118.83^*^ | 2.72 | 127.33 | 124.02^*^ | 3.31 | 0.59 |
|  | 8+ | 127.12 | 124.64^*^ | 2.48 | 133.73 | 131.03^*^ | 2.70 | 0.22 |  | 126.50 | 123.79^*^ | 2.71 | 132.51 | 129.52^*^ | 2.99 | 0.29 |
|  | 9+ | 132.35 | 129.28^*^ | 3.08 | 139.25 | 136.47^*^ | 2.79 | -0.29 |  | 132.06 | 129.12^*^ | 2.94 | 138.66 | 135.19^*^ | 3.48 | 0.54 |
|  | 10+ | 136.95 | 133.94^*^ | 3.01 | 144.61 | 141.15^*^ | 3.46 | 0.45 |  | 137.80 | 134.73^*^ | 3.07 | 145.10 | 141.29^*^ | 3.82 | 0.75 |
|  | 11+ | 142.17 | 138.94^*^ | 3.24 | 150.62 | 146.31^*^ | 4.32 | 1.08 |  | 144.44 | 140.99^*^ | 3.45 | 151.68 | 147.08^*^ | 4.60 | 1.15 |
|  | 12+ | 147.48 | 143.41^*^ | 4.07 | 157.80 | 151.45^*^ | 6.35 | 2.28 |  | 149.63 | 145.75^*^ | 3.87 | 156.01 | 151.24^*^ | 4.78 | 0.90 |
|  | 13+ | 156.26 | 151.44^*^ | 4.82 | 164.34 | 159.93^*^ | 4.42 | -0.40 |  | 154.99 | 152.20^*^ | 2.80 | 158.85 | 155.77^*^ | 3.08 | 0.28 |
|  | 14+ | 162.13 | 158.20^*^ | 3.93 | 169.70 | 165.11^*^ | 4.59 | 0.67 |  | 156.97 | 154.67^*^ | 2.30 | 160.21 | 157.89^*^ | 2.33 | 0.02 |
|  | 15+ | 166.57 | 163.38^*^ | 3.19 | 172.04 | 168.75^*^ | 3.28 | 0.09 |  | 157.98 | 155.89^*^ | 2.09 | 160.93 | 158.48^*^ | 2.45 | 0.36 |
|  | 16+ | 169.10 | 166.48^*^ | 2.62 | 173.28 | 170.53^*^ | 2.75 | 0.13 |  | 158.78 | 157.06^*^ | 1.72 | 161.23 | 159.17^*^ | 2.06 | 0.34 |
|  | 17+ | 170.50 | 168.10^*^ | 2.39 | 173.62 | 171.65^*^ | 1.97 | -0.42 |  | 159.28 | 157.56^*^ | 1.71 | 161.37 | 159.39^*^ | 1.98 | 0.27 |
|  | 18+ | 170.95 | 168.53^*^ | 2.41 | 173.70 | 171.40^*^ | 2.31 | -0.11 |  | 159.33 | 157.22^*^ | 2.11 | 161.17 | 159.21^*^ | 1.97 | -0.14 |
|  | Average |  |  | 3.15 |  |  | 3.46 | 0.31 |  |  |  | 2.62 |  |  | 3.07 | 0.45 |
| Rural | 7+ | 119.01 | 116.72^*^ | 2.29 | 126.54 | 122.07^*^ | 4.46 | 2.17 |  | 117.86 | 115.89^*^ | 1.97 | 125.45 | 120.78^*^ | 4.67 | 2.70 |
|  | 8+ | 123.42 | 121.06^*^ | 2.36 | 131.90 | 127.15^*^ | 4.75 | 2.39 |  | 122.54 | 120.13^*^ | 2.40 | 130.91 | 126.06^*^ | 4.85 | 2.45 |
|  | 9+ | 128.35 | 125.65^*^ | 2.70 | 136.91 | 131.85^*^ | 5.06 | 2.36 |  | 127.68 | 124.91^*^ | 2.77 | 136.58 | 131.16^*^ | 5.42 | 2.65 |
|  | 10+ | 133.00 | 130.44^*^ | 2.56 | 142.06 | 136.84^*^ | 5.22 | 2.66 |  | 132.83 | 130.12^*^ | 2.70 | 142.89 | 137.23^*^ | 5.65 | 2.95 |
|  | 11+ | 137.64 | 134.79^*^ | 2.85 | 147.32 | 142.15^*^ | 5.17 | 2.32 |  | 138.82 | 135.69^*^ | 3.12 | 148.85 | 142.97^*^ | 5.88 | 2.76 |
|  | 12+ | 142.36 | 139.35^*^ | 3.01 | 153.71 | 147.61^*^ | 6.11 | 3.10 |  | 144.50 | 141.22^*^ | 3.28 | 153.51 | 148.15^*^ | 5.36 | 2.08 |
|  | 13+ | 150.58 | 146.76^*^ | 3.83 | 161.36 | 154.83^*^ | 6.52 | 2.70 |  | 151.15 | 148.40^*^ | 2.75 | 157.08 | 152.69^*^ | 4.39 | 1.64 |
|  | 14+ | 156.23 | 153.14^*^ | 3.09 | 166.65 | 161.02^*^ | 5.64 | 2.55 |  | 153.37 | 151.44^*^ | 1.93 | 158.28 | 155.23^*^ | 3.05 | 1.12 |
|  | 15+ | 161.13 | 158.72^*^ | 2.42 | 169.91 | 165.38^*^ | 4.53 | 2.12 |  | 154.90 | 153.49^*^ | 1.41 | 159.32 | 156.44^*^ | 2.87 | 1.46 |
|  | 16+ | 164.91 | 163.03^*^ | 1.88 | 171.84 | 168.15^*^ | 3.68 | 1.80 |  | 155.77 | 154.59^*^ | 1.19 | 159.50 | 157.17^*^ | 2.33 | 1.14 |
|  | 17+ | 166.94 | 165.15^*^ | 1.79 | 172.45 | 169.23^*^ | 3.21 | 1.43 |  | 156.49 | 155.34^*^ | 1.15 | 159.97 | 157.56^*^ | 2.40 | 1.25 |
|  | 18+ | 167.65 | 166.10^*^ | 1.54 | 172.17 | 169.87^*^ | 2.30 | 0.75 |  | 156.90 | 155.60^*^ | 1.29 | 159.71 | 157.57^*^ | 2.14 | 0.85 |
|  | Average |  |  | 2.53 |  |  | 4.72 | 2.20 |  |  |  | 2.16 |  |  | 4.09 | 1.92 |
|  |  |  |  |  |  |  |  |  |  |  |  |  |  |  |  |  |
|  |  |  |  |  |  |  |  |  |  |  |  |  |  |  |  |  |
|  |  |  |  |  |  |  |  |  |  |  |  |  |  |  |  |  |

T-test: western compared with eastern subjects, *P<0.001;

d1:height difference (eastern -western) in 1985;

d2: height difference( eastern -western )in 2010
