# Supplementary material for: Height inequalities and their change trends in China during 1985–2010: results from 6 cross-sectional surveys on children and adolescents aged 7–18 years
Source: BMC Public Health. 2017 May 18;17:473. doi: 10.1186/s12889-017-4402-9 (PMC5437404; doi:10.1186/s12889-017-4402-9)
Supplement: Supplementary file 4 — Height of Shanghai and Guizhou subjects and Shanghai-Guizhou height differences in 1985 and 2010 (DOCX 22 kb). [file 12889_2017_4402_MOESM4_ESM.docx]

Additional file 4: Table S4. Height of Shanghai and Guizhou subjects and Shanghai-Guizhou height differences in 1985 and 2010(cm)

|  |  |  |  | Male |  |  |  |  |  |  |  | Female |  |  |  |  |
| --- | --- | --- | --- | --- | --- | --- | --- | --- | --- | --- | --- | --- | --- | --- | --- | --- |
| Urban | Age |  | 1985 |  |  | 2010 |  | d2-d1 |  |  | 1985 |  |  | 2010 |  | d2-d1 |
|  |  | Shanghai | Guizhou | d1 | Shanghai | Guizhou | d2 |  |  | Shanghai | Guizhou | d1 | Shanghai | Guizhou | d2 |  |
|  | 7+ | 123.54 | 117.81^*^ | 5.73 | 129.17 | 123.39^*^ | 5.78 | 0.05 |  | 122.18 | 117.81^*^ | 4.78 | 128.10 | 121.53^*^ | 6.57 | 1.79 |
|  | 8+ | 129.03 | 122.39^*^ | 6.64 | 134.36 | 127.33^*^ | 7.03 | 0.39 |  | 128.07 | 122.39^*^ | 5.93 | 133.01 | 126.79^*^ | 6.22 | 0.29 |
|  | 9+ | 133.94 | 126.55^*^ | 7.39 | 139.55 | 133.01^*^ | 6.54 | -0.85 |  | 133.67 | 126.55^*^ | 6.30 | 139.25 | 132.99^*^ | 6.26 | -0.04 |
|  | 10+ | 138.35 | 131.03^*^ | 7.32 | 143.77 | 136.30^*^ | 7.47 | 0.15 |  | 139.21 | 131.03^*^ | 7.15 | 145.56 | 137.83^*^ | 7.73 | 0.58 |
|  | 11+ | 144.11 | 135.92^*^ | 8.19 | 151.73 | 141.49^*^ | 10.24 | 2.05 |  | 146.12 | 135.92^*^ | 6.94 | 152.82 | 142.33^*^ | 10.49 | 3.55 |
|  | 12+ | 149.37 | 140.35^*^ | 9.02 | 157.79 | 148.97^*^ | 8.82 | -0.20 |  | 150.69 | 140.35^*^ | 7.44 | 156.73 | 148.41^*^ | 8.32 | 0.88 |
|  | 13+ | 157.96 | 148.40^*^ | 9.56 | 164.59 | 156.05^*^ | 8.54 | -1.02 |  | 155.79 | 148.40^*^ | 6.03 | 159.19 | 152.37^*^ | 6.82 | 0.79 |
|  | 14+ | 164.31 | 155.36^*^ | 8.95 | 170.91 | 160.49^*^ | 10.42 | 1.47 |  | 157.85 | 155.36^*^ | 6.17 | 160.82 | 154.99^*^ | 5.83 | -0.34 |
|  | 15+ | 168.36 | 160.97^*^ | 7.39 | 172.68 | 163.73^*^ | 8.95 | 1.56 |  | 158.81 | 160.97^*^ | 4.84 | 162.45 | 155.04^*^ | 7.41 | 2.57 |
|  | 16+ | 171.00 | 164.37^*^ | 6.63 | 174.76 | 166.34^*^ | 8.42 | 1.79 |  | 159.36 | 164.37^*^ | 4.28 | 162.55 | 155.89^*^ | 6.66 | 2.38 |
|  | 17+ | 171.25 | 165.29^*^ | 5.96 | 174.93 | 166.97^*^ | 7.96 | 2.00 |  | 159.87 | 165.29^*^ | 4.99 | 161.39 | 156.73^*^ | 4.66 | -0.33 |
|  | 18+ | 172.52 | 165.83^*^ | 6.42 | 175.01 | 167.02^*^ | 7.99 | 1.57 |  | 159.74 | 165.83^*^ | 5.10 | 162.72 | 156.45^*^ | 6.27 | 1.17 |
|  | Average |  |  | 7.43 |  |  | 8.18 | 0.75 |  |  |  | 5.83 |  |  | 6.94 | 1.11 |
| Rural | 7+ | 118.96 | 114.83^*^ | 4.13 | 127.25 | 119.25^*^ | 8.00 | 3.87 |  | 118.24 | 113.64^*^ | 4.60 | 126.46 | 118.58^*^ | 7.88 | 3.28 |
|  | 8+ | 123.31 | 118.05^*^ | 5.26 | 133.21 | 124.64^*^ | 8.57 | 3.31 |  | 122.32 | 117.48^*^ | 4.83 | 132.50 | 123.41^*^ | 9.09 | 4.26 |
|  | 9+ | 128.57 | 122.31^*^ | 6.26 | 137.66 | 127.78^*^ | 9.88 | 3.62 |  | 128.51 | 122.03^*^ | 6.48 | 137.84 | 128.12^*^ | 9.72 | 3.24 |
|  | 10+ | 133.11 | 126.73^*^ | 6.38 | 142.83 | 133.57^*^ | 9.26 | 2.88 |  | 133.66 | 126.11^*^ | 7.55 | 143.80 | 134.57^*^ | 9.23 | 1.68 |
|  | 11+ | 137.68 | 130.53^*^ | 7.15 | 148.38 | 138.52^*^ | 9.86 | 2.71 |  | 139.97 | 131.50^*^ | 8.47 | 151.30 | 139.64^*^ | 11.66 | 3.19 |
|  | 12+ | 143.57 | 135.52^*^ | 8.05 | 156.44 | 143.54^*^ | 12.90 | 4.85 |  | 146.68 | 136.67^*^ | 10.01 | 155.51 | 144.26^*^ | 11.25 | 1.24 |
|  | 13+ | 151.24 | 143.67^*^ | 7.57 | 163.54 | 149.95^*^ | 13.59 | 6.02 |  | 152.02 | 145.34^*^ | 6.68 | 158.03 | 149.35^*^ | 8.68 | 2.00 |
|  | 14+ | 156.47 | 149.71^*^ | 6.76 | 168.30 | 155.55^*^ | 12.75 | 5.99 |  | 154.19 | 148.11^*^ | 6.08 | 159.24 | 152.03^*^ | 7.21 | 1.13 |
|  | 15+ | 162.18 | 154.19^*^ | 7.99 | 172.13 | 159.99^*^ | 12.14 | 4.15 |  | 155.57 | 150.07^*^ | 5.50 | 161.50 | 152.94^*^ | 8.56 | 3.06 |
|  | 16+ | 165.52 | 159.43^*^ | 6.09 | 174.70 | 164.22^*^ | 10.48 | 4.39 |  | 156.36 | 151.15^*^ | 5.21 | 161.32 | 152.91^*^ | 8.41 | 3.20 |
|  | 17+ | 167.52 | 161.16^*^ | 6.36 | 173.93 | 164.28^*^ | 9.65 | 3.29 |  | 156.83 | 152.24^*^ | 4.59 | 161.10 | 153.41^*^ | 7.69 | 3.10 |
|  | 18+ | 168.76 | 162.75^*^ | 6.01 | 173.92 | 165.62^*^ | 8.30 | 2.29 |  | 157.04 | 153.04^*^ | 4.00 | 161.08 | 153.75^*^ | 7.33 | 3.33 |
|  | Average |  |  | 6.50 |  |  | 10.45 | 3.95 |  |  |  | 6.17 |  |  | 8.89 | 2.73 |
|  |  |  |  |  |  |  |  |  |  |  |  |  |  |  |  |  |

T-test: Guizhou compared with Shanghai subjects, *P<0.001;

d1:height difference( Shanghai -Guizhou )in 1985;

d2: height difference(Shanghai -Guizhou )in 2010
